# Quality of Drinking-water at Source and Point-of-consumption—Drinking Cup As a High Potential Recontamination Risk: A Field Study in Bolivia

**DOI:** 10.3329/jhpn.v28i1.4521

**Published:** 2010-02

**Authors:** Simonne Rufener, Daniel Mäusezahl, Hans-Joachim Mosler, Rolf Weingartner

**Affiliations:** ^1^ Group of Hydrology, Geographical Institute, University of Bern, Hallerstrasse 12, 3012 Bern, Switzerland; ^2^ Department of Public Health and Epidemiology, Swiss Tropical Institute, Socinstrasse 57, PO Box, CH-4002 Basel, Switzerland; ^3^ Swiss Federal Institute of Aquatic Sciences and Technology, EAWAG, Überlandstrasse 133, PO Box 611, 8600 Dübendorf, Switzerland

**Keywords:** Disinfection, Drinking-water, Hygiene, Water management, Water pollution, Water supply, Bolivia

## Abstract

In-house contamination of drinking-water is a persistent problem in developing countries. This study aimed at identifying critical points of contamination and determining the extent of recontamination after water treatment. In total, 81 households were visited, and 347 water samples from their current sources of water, transport vessels, treated water, and drinking vessels were analyzed. The quality of water was assessed using *Escherichia coli* as an indicator for faecal contamination. The concentration of *E. coli* increased significantly from the water source [median=0 colony-forming unit (CFU)/100 mL, interquartile range (IQR: 0–13)] to the drinking cup (median=8 CFU/100 mL; IQR: 0–550; n=81, z=−3.7, p<0.001). About two-thirds (34/52) of drinking vessels were contaminated with *E. coli*. Although boiling and solar disinfection of water (SODIS) improved the quality of drinking-water (median=0 CFU/100 mL; IQR: 0–0.05), recontamination at the point-of-consumption significantly reduced the quality of water in the cups (median=8, IQR: 0–500; n=45, z=−2.4, p=0.015). Home-based interventions in disinfection of water may not guarantee health benefits without complementary hygiene education due to the risk of post-treatment contamination.

## INTRODUCTION

Every year, some 1.6 million people die due to diarrhoea because of contaminated drinking-water (1). In developing countries, a majority of households are still deprived of running water; hence, drinking-water must be collected at source, which is often located many hundreds of metres away from home and transported to the household where it is stored until consumption. Researchers have repeatedly observed that the microbiological quality of water in transportation and drinking vessels in the home is lower than that at the source, suggesting that contamination may occur at different stages during the process from collection of water to consumption (2–5). Bacterial counts in water at source and water stored in the household showed that the contamination is greater in cases where the faecal coliform counts in the water at source are low (4). Consequently, in-house contamination may reverse the health benefits that are gained by improvements in community water supply.

The practice of open storage of drinking-water allows for faecal contamination to occur inside the household. Contamination by hands and domestic animals has been shown to be the predominant causes of declining the quality of water (6, 7). This pattern has been confirmed by subsequent studies of water contamination in rural Sierra Leone, rural Honduras, South Africa, and Zimbabwe (5, 8). While the detrimental effects of in-house contamination are known, the exact point of contamination remains still unclear.

**Fig. 1.**
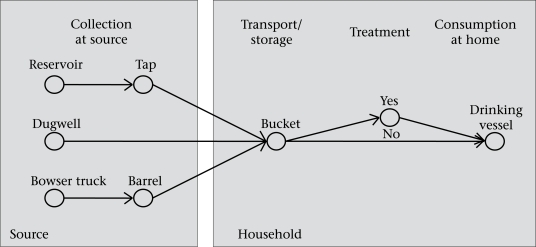
Potential contamination pathway between source of water supply and point-of-consumption

The main goal of the present study was to locate intrinsic and specific points where faecal contamination may occur in the process from the point-of-collection to the point-of-use. Therefore, we measured the quality of water at all stages along the potential contamination pathway from the water source to the drinking cups used in the household (Fig. 1). Although water can be collected at different water sources, our study focused on water collected from reservoirs, dugwells, or bowser trucks. Subsequently, the water is ultimately transported, may be stored at home in a bucket, and after eventual treatment put in a drinking vessel before consumption. Each of these points within the pathway from source to mouth was investigated.

Circles represent sampling points

## MATERIALS AND METHODS

### Study area and population

For this study, participating households were situated in three different geographical regions, i.e. in the highlands (ca. 3750 m.a.s.l.), the valley (ca. 2600 m.a.s.l.), and the lowlands (ca. 250 m.a.s.l.) and in different levels of urbanization (i.e. periurban and rural). The two selected study villages of the highlands were situated in the community of Uncía, i.e. Lawa Lawa and Cotaviri. The three selected sampling sites for the study area in the valley, i.e. Calicantu, Valle Hermoso, and Kara Kara, were situated in the southern zone of Cochabamba, and the one village selected in the lowland, i.e. Núcleo 24, is part of the San Julian district of Santa Cruz. All regions were previously exposed to health and hygiene-promotion campaigns implemented by different civil society organizations and governmental entities. In addition, an ongoing solar water disinfection (SODIS)-promotion campaign had started in all the communities six months before we conducted the study. The SODIS campaign was part of a project comparing different household water-treatment promotion strategies (9).

In rural communities, every second household was systematically selected. In peri-urban areas, participating households were selected by the random-route method (10). Specifically, streets within neighbourhoods were randomly selected, and every third house was enrolled in the study. The nature and purpose of the study were outlined in an initial community group meeting and individually explained in detail to household members during enrollment.

Participants provided verbal consent for their participation in the study. We sampled 27 rural households in the highlands, 25 rural households in the lowland, and 29 semi-urban households in the valley. Only two of the 83 households that were approached refused to participate.

### Collection of water samples

During unannounced visits to each of the 81 participating households, water samples were collected from their drinking-water sources, transport vessels, treated water, and a drinking cup (Fig. 1). The term ‘drinking cup’ is subsequently used in the text describing all kinds of vessels used for actual drinking. In most (89%) households, transport vessels also served as storage vessels. In total, 347 microbiological analyses were immediately conducted on-site using a membrane-filtration method, i.e. the Oxfam DelAgua® water testing-kit (11). The quality of water was quantitatively assessed through the enumeration of colony-forming units (CFUs) of *Escherichia coli*, which was used as an indicator organism for faecal contamination. Membrane filters were incubated on mFC agar at 44±0.5 °C for 18–24 hours. Negative controls, i.e. 100 mL of sterile distilled water, were processed after every twentieth sample to ensure that the equipment had been adequately sanitized.

Water samples were collected according to the standard method described in the guidelines of the World Health Organization for the quality of drinking-water (12). Water samples from bowser trucks were collected in a sterile sample-bottle, stored on ice, and analyzed within four hours. Only unflamed samples were taken in an effort to represent the normal water quality that would be accessible by the families (5).

**Socioeconomic and behavioural data**

Water, sanitation practices, and demographic data were collected through a questionnaire that contained structured questions and was administered by trained local interviewers in Spanish or Quechua. The questions were related to water-extraction patterns, type of water transport, water-treatment methods and cleaning habits, type and material of water-related issues, and sanitation facilities. The person interviewed, who was the mother and/or housewife in most cases, was the individual responsible for the management of drinking-water in the household.

### Statistical analyses

To account for the zero inflated and right censored distribution of the number of CFUs, only non-parametric methods were applied in analyses of data. The differences between related water samples, i.e. between different sampling points within individual households, were assessed with the Wilcoxon signed rank test. Data were log-transformed to meet the assumption of symmetry. In the case of independent samples, i.e. comparisons between groups of households with different water-treatment behaviours, the Mann-Whitney-U-test was applied. The relationships between variables have been determined by partial Spearman rank analysis. All analyses were performed using the SPSS software (version 10.0).

## RESULTS

### Demographic data and housing characteristics

In almost half (43%) of the families visited, each comprised 5–6 members within the household, and 55% of the families had at least one child aged less than five years. The highest level of education for the participants was observed in the study area in the valley, with a median of seven (IQR: 1.25–12) years of school attendance compared to a median of five (IQR: 3–8) years of schooling in the lowland and a median of three (IQR: 0–3) years in the highland. The median number of rooms per household was 3 (IQR: 2–4), and 63% of the homes had a natural soil-floor. Most (95%) households owned domestic animals. In total, 37 households raised pigs; additionally, 25 households raised chicken; and 16 households owned cows.

### Types of water sources and home-based water-management practice

The three geographical regions featured different water sources compared to one another; all the water sources were typical for Bolivia. In the lowland, piped and tapped water systems, and traditional dugwells with hand-pumps at the home were predominantly used. In the valley, bowser trucks distributed drinking-water. In the highland, piped systems with taps in the household-yard prevailed (Table 1). Overall, community-based pipe-tap systems were most frequently used (58%). Bowser trucks as a water source served 36% of the households in the study area. Sanitary inspections that were completed using procedures based on a manual developed by the World Health Organization (12) indicated that all the sources monitored were classified as being at ‘intermediate’ to ‘very high’ levels of risk for contamination.

**Table 1.** Water-management practices in sample households

Household characteristics	All households		Highland	Valley	Lowland
	% (100%)	No. (n=81)	No. (n=27)	No. (n=29)	No. (n=25)
Water source					
Dugwell (home-based)	6 (5)	5 (4)	0	0	5
Pipe-tap system (home-based)	58 (22)	47 (18)	27	0	20
Bowser truck	36	29	0	29	0
Type of transport/storage vessel					
Bucket (covered)	56 (5)	45 (4)	26	9	10
Barrel (covered)	22 (10)	18 (8)	0	17	1
Canister (covered)	14 (3)	11 (2)	0	3	8
Others (covered)	5 (0)	4 (0)	1	0	3
Not recorded	3	3	0	0	3
Cleaning of transport/storage vessel					
Yes	53	43	24	19	0
At least daily	31	25	10	15	0
Half-weekly	4	3	0	3	0
Weekly	18	15	14	1	0
No	47	38	3	10	25
Cleaning of drinking cup					
Yes	96	79	26	28	25
At least daily	48	39	23	12	4
Half-weekly	31	25	0	9	16
Weekly	17	14	3	6	5
No	4	2	1	1	0
Type of treated water available during household visit					
Raw water only	44	36	18	8	10
Boiled water	19	15	0	15	0
Solar water disinfection	37	30	9	6	15

Of the households visited, 44% used plastic-cups for drinking, and 24% used cups made of tin or other metals. Glasses or glass-cups were used in 11% of the homes. Buckets, barrels, and canisters were mainly used as transport vessels. Only 14 of the 78 observed transport vessels were covered with a lid. Approximately half (42%) of the participating households cleaned their transport vessel with a detergent, and only 31% of those that used a detergent did so at least daily. Drinking cups were washed in 96% of the participating households. Only 48% of the families cleaned these at least once a day. About 70% of the interviewed families treated water before consumption. During unannounced visits by the project staff, 45 of 57 households that treated their drinking-water were able to show SODIS bottles or previously boiled water ready to drink in the home. The remaining 36 households had only raw water available in their homes.

### Water-quality between supply source and point-of-consumption

**Fig. 2.**
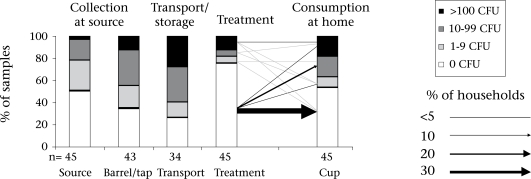
Water samples along the transmission pathway

**Fig. 3.**
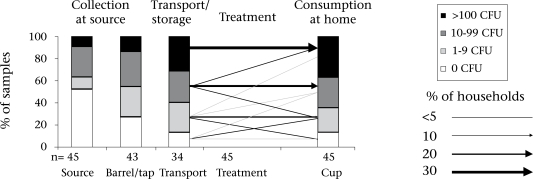
Water samples along the transmission pathway

The quality of drinking-water deteriorated steadily along the pathway from the supply source to the drinking cup (Table 2, Fig. 2 and 3). The median concentration of *E. coli* increased significantly from 0 CFU/100 mL (IQR: 0–13) at the source to 8 CFU/100 mL (IQR: 0–550) at the point-of-consumption in the home (Wilcoxon signed rank test: n=81, Z=−3.7, p<0.001).

**Table 2.** Water-quality at source and drinking cup

Group	Source	Barrel/tap	Storage-container	Treatment-container	Drinking vessel
All households	0 (0–13)	7 (0–49)	15 (2–450)	0 (0–1)	8 (0–550)
Home-based water treatment	0 (0–5)	8 (0–45)	23 (0–360)	0 (0–1)	0 (0–34)
No home-based water treatment	0 (0–37)	6 (0–66)	13 (3–510)	NA	34 (4–2,400)

NA=Not applicable;

Figures indicate median contamination in CFU/100 mL and interquartile range in parentheses

The thicknesses of the arrows are proportional to the contamination with *E. coli* (CFU/100 mL). Only households that were applying a water-treatment method are included. Categories of *E. coli* contamination are based on the WHO criteria (12). Two households with dugwell water supplies, barrel, or tap-samples were not collected. Eleven households did not use a transport vessel;
CFU=Colony-forming unit;
WHO=World Health Organization

The thicknesses of the arrows are proportional to the contamination with *E. coli* (CFU/100 mL). Only households that were not applying any water-treatment method are included. Categories of *E. coli* contamination are based on guidelines by the WHO (12). Three households with dugwell water supplies, barrel, or tap-samples were not collected. Six households did not use a transport vessel;
CFU=Colony-forming unit;
WHO=World Health Organization

After the transportation of water, home-based water treatments reduced the median concentration to 0 CFU/100 mL (IQR: 0–1); however, recontamination at the point-of-consumption significantly reduced the quality of water in the cups (median=8, IQ=0–500; n=45, z=−2.4, p=0.015). Only 36% of the treated water samples were free from *E. coli.*

### Home-based water treatment

About 56% of the households treated their water by boiling or with SODIS. None of the households chlorinated their drinking-water. In one of 15 households that practised boiling, water samples collected from the treatment devices, i.e. the cooking pot, still contained *E. coli*, and in 10 of 30 households that practised SODIS, water samples from the treatment devices still contained *E. coli*. According to the observations of the interviewers, the failure observed in the SODIS procedure was due to insufficient exposure of the bottles to sunlight.

Of the 34 clean treatment samples, 65% remained clean; however, recontamination occurred in the remaining 35% at the point-of-consumption (Fig. 3). Regardless of the recontamination that occurred at the point-of-consumption and the occasional failures that occurred in applying a treatment method, the median contamination of water from drinking cups at households not treating drinking-water was more than four times higher (median=34 CFU/100 mL; IQR: 4–2,400) than that at households treating their drinking-water (median=0 CFU/100 mL; IQR: 0–43; Mann-Whitney-U-test: n=81, z=3.3, p=0.001).

### Correlation of water-quality at source and in drinking cups

**Fig. 4.**
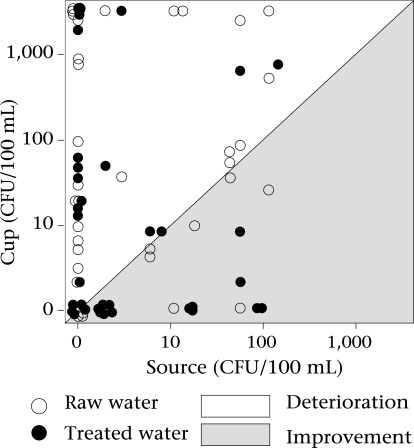
Effect of water-quality at source on water-quality in drinking cup stratified by water-treatment method

We detected no significant relationship between the quality of drinking-water at the source and the quality of water in drinking cups within the participating households (n=79, partial Spearman rank correlation coefficient=−0.01, p=0.94, adjusted for water-treatment behaviour). The result indicates that pathogen-free water at the source is not a guarantee for safe and pathogen-free drinking-water at the point-of-consumption (Fig. 4).

Small random noise was added to avoid over plotting. No correlation was found between quality of drinking-water at the supply source and the quality of drinking-water in drinking cup (n=79, Spearman rank correlation coefficient=−0.01, p=0.94);
CFU=Colony-forming unit

## DISCUSSION

This study investigated potential contamination of drinking-water along the domestic pathway from collection to consumption, including the point-of-collection at the main water source, during transportation, in the storage at the home, and at the point-of-consumption. We found that faecal contamination was low at the water source but increasingly deteriorated throughout storage and treatment within the households. Although home-based water treatment improved the quality of water immediately, the quality frequently worsened again in the drinking cups, thereby reflecting a recontamination just before drinking.

Several potential limitations should be considered in the interpretation of the findings of the study. The participating households were heterogeneous in terms of geographical location and source of drinking-water. The size of our study sample did not allow for stratified analyses. Despite this heterogeneity, the potential areas for contamination along the pathway from collection to consumption are generally comparable. Moreover, *E. coli* might not be an adequate indicator for faecal contamination (13). To allow us to compare our findings with similar studies, we used *E. coli* as an indicator bacterium and applied the DelAgua® membrane-filtration method because it is a universally-accepted and most practical method for detecting waterborne coliforms on-site.

Results of previous studies are in agreement with our findings that the quality of water may significantly deteriorate after collection; i.e. contamination occurs in transport vessels (4, 5) or in the household domain in general (6, 8, 14). However, a recent publication found lower concentrations of *Enterococci* and *E. coli* in water stored at home compared to that at the source (15). Inadequate cleanliness of storage and transport-containers has been described as a key source of drinking-water contamination in many settings worldwide (8, 16–19). One possible cause for the contamination of previously safe water may be the presence of biofilms on the inner surfaces of containers, which emphasizes the need for repeated cleaning (20). In our study, only 31% of the participating households cleaned their transport vessel daily, and most (88%) used uncovered buckets as transport vessels. The use of narrow-mouthed containers to prevent contamination (21) was uncommon among our sample.

Recontamination of drinking-water in the drinking cup was observed in 35% of the participating households. In another study, the researchers found that boiled water was more frequently contaminated when served in a drinking cup compared to water taken directly from a storage container containing boiled water, which supports our finding (22). Therefore, water at the point-of-consumption cannot always be considered safe, despite previous, effective water treatment, such as boiling or SODIS.

Additionally, the findings of different studies, including ours, emphasize the importance of reducing the risk of contaminating drinking-water just before use. Hygiene measures, such as cleaning of drinking cup, could reduce this risk; moreover, residuals after chlorination could still be active in drinking vessel. However, while cleaning of drinking vessels and water management with clean hands are effective hygiene interventions (22), no studies have been published that had investigated the decontaminating effects of residual chlorine in pouring water and drinking-water. Even if such an effect of residual chlorine in drinking glass exists, chlorination was not common in our study area.

The scope of our project did not allow us to investigate the exact source of contamination at the point-of-consumption, specifically distinguishing between dirty hands and dirty cups as the cause of contamination. Dirty hands may contaminate water not only through handling during collection and transportation (4, 7, 23) but also when handling drinking vessels or scooping drinking-water from storage vessels (22, 24–26). One investigation further showed that 91% of 93 hand-rinsing water samples contained a geometric mean of 177 *E. coli* CFU/100 mL and that hand-rinse colony counts correlated directly with cup-rinse water-colony counts in the same household (22). Therefore, hygiene education with regard to water management and hygiene practices, such as cleanliness of the home (including cleaning of cups and buckets and food preparation and storage), is of paramount importance in the prevention of childhood diarrhoea (24, 25, 27, 28). Safe water-handling and storage practices can be promoted with little investment from households (7). Water-supply programmes at the community level should focus more on sanitation practices at the point-of-consumption.

## ACKNOWLEDGEMENTS

This research was partially funded by the Commission for Research Partnerships with Developing Countries (KFPE), Migros, Bawaco AG, the Swiss Society for Hydrology and Limnology, and the Swiss Society for Applied Geography. The authors are grateful to the inhabitants of the study villages and the members of the water committees for their cooperation. The teamwork and support from the Fundación SODIS and the Centro de Aguas y Saneamiento Ambiental (CASA) in Cochabamba during data collection is greatly appreciated. The authors also thank Jan Hattendorf for his statistical advice and writing support.
